# Conformational tuning of a DNA-bound transcription factor

**DOI:** 10.1093/nar/gkz291

**Published:** 2019-04-25

**Authors:** Giuseppe Sicoli, Hervé Vezin, Karin Ledolter, Thomas Kress, Dennis Kurzbach

**Affiliations:** 1 Univ. Lille, CNRS, UMR 8516 - LASIR - Laboratoire de Spectrochimie Infrarouge et Raman, F-59000 Lille, France; 2 University Vienna, Department for Structural and Computational Biology, Max F. Perutz Laboratories, Campus Vienna BioCenter 5, 1030 Vienna, Austria; 3 Laboratoire des biomolécules, LBM, Département de chimie, École normale supérieure, PSL University, Sorbonne Université, CNRS, 75005 Paris, France; 4 University of Vienna, Faculty of Chemistry, Institute of Biological Chemistry, Währinger Str. 38, 1090 Vienna, Austria

## Abstract

Transcription factors are involved in many cellular processes that take place remote from their cognate DNA sequences. The efficiencies of these activities are thus in principle counteracted by high binding affinities of the factors to their cognate DNAs. Models such as facilitated diffusion or dissociation address this apparent contradiction. We show that the MYC associated transcription factor X (MAX) undergoes nanoscale conformational fluctuations in the DNA-bound state, which is consistent with facilitated dissociation from or diffusion along DNA strands by transiently reducing binding energies. An integrative approach involving EPR, NMR, crystallographic and molecular dynamics analyses demonstrates that the N-terminal domain of MAX constantly opens and closes around a bound DNA ligand thereby dynamically tuning the binding epitope and the mode of interaction.

## INTRODUCTION

The regulation of cellular machinery relies upon interactions of DNA with a plethora of transcription factors (TFs) (1–3). Although the understanding of DNA–TF recognition is of widespread medicinal, pharmacological, and biological interest, many essential features of these interactions remain poorly understood despite long-standing research efforts. It has, for example, become increasingly evident that the classical model of nuclear receptors (NR) as rigid multidomain TFs cannot explain their variable activities or their different responses to various ligands (4). Instead, a model of ‘functional intrinsic disorder’—i.e. the presence of physiologic activity despite a lack of stable secondary and tertiary structures—must be invoked to describe this class of TFs (4). In such ensemble-based models, the internal dynamics and intrinsic flexibility of TFs are used to explain, for example, how various intrinsically disordered domains can elicit different allosteric responses upon ligand binding. These insights allow one to explain how different ligand interactions can guide TF functionality. Further evidence focusing in particular on the structural fluctuations within TF/DNA-binding interfaces was recently reported, highlighting the relation between structural dynamics and the high activities of TFs. Indeed, backbone dynamics as well as transiently formed contacts between TF side-chains and the target DNAs can influence and optimize the DNA recognition sequence. (5)

The better understanding of the dynamics of TFs gave rise to recent approaches that attempt to rationalize experimentally observed fast DNA transcriptional rates and interactions by answering to a key question, which is often summarized under the so-called ‘speed-stability paradox’: How do TFs fulfil their well-timed functions in solution despite strong binding affinities to their cognate DNAs? (6). Several models have recently been proposed to address this apparent contradiction, based on phenomenological descriptions of either facilitated diffusion of a non-specifically bound TF along DNA strands (7,8), or partial unbinding of a TF–DNA complex to expose the binding site to competitors (facilitated dissociation) (9). These models provide a basis for physiological activity of TFs remote from their recognition motifs or their target DNAs, respectively.

A prominent example of the speed-stability paradox is the MYC associated factor X (MAX). MAX occurs physiologically as a rigid coiled-coil homodimer (here denoted MAX_2_) bound tightly to its cognate DNA sequence (CACGTG, denoted as EBOX motif). To develop transcriptional activity, MAX_2_ needs to dissociate into monomers and subsequently heterodimerize with its partner molecule MYC to form the MYC:MAX complex. Evidently, this vital process must be preceded by dissociation of MAX_2_ from the EBOX DNA. However, a very high MAX_2_-DNA binding affinity would in principle counteract the dissociation event, and alternative explanatory concepts such as facilitated dissociation are therefore needed to enlighten MAX_2_’s activity.

We here contribute to the rationalization of MAX_2_’s biological activity by demonstrating that nanoscale conformational fluctuations in its DNA-bound state can serve as a structural basis for facilitated diffusion as well as facilitated dissociation, which in turn might facilitate MYC:MAX heterodimerization (10). Indeed, the DNA-binding epitope of MAX_2_ is shown to open and close around the EBOX DNA ligand giving rise to conformational fluctuations that are likely to assist dissociation from the DNA strand.

## MATERIALS AND METHODS

### NMR

^1^H–^15^N transverse relaxation optimized spectroscopy (TROSY) for PRE measurements was recorded at 20°C using a Bruker HDIII wide-bore 800 MHz spectrometer. Spectra were recorded in the States-TPPI/PFG mode for quadrature detection with carrier frequencies for ^1^H^N^ and ^15^N of 4.73 and 120.0 ppm, respectively. The samples contained 0.4 mM MAX, 25 mM MES, and 25 mM NaCl (pH 5.5) in a 90% H_2_O/10% D_2_O mixture.

All NMR spectra were processed and analyzed using NMRPipe and SPARKY. (11,12) A squared and 60° phase-shifted sine bell window function was applied in all dimensions for apodization. Time domain data were zero-filled to twice the data set size, prior to Fourier transformation. ^1^H–^15^N cross peak assignments were obtained from the biological magnetic resonance data base (BMRB) entry 5956 and the work by Sauvé *et al.* (13)

### EPR

DEER experiments were performed on a Bruker ELEXSYS E580 at 50 K operating at Q-band frequency (34 GHz), using a four-pulse sequence:

\begin{eqnarray*} &&{\rm{\pi }}/2({{\rm{\nu }}_1}) - {{\rm{\tau }}_1} - {\rm{\pi }}({{\rm{\nu }}_1}) - {{\rm{\tau }}_0} - {\rm{\pi }}({{\rm{\nu }}_2}{\rm{)}} - {\rm{(}}{{\rm{\tau }}_{\rm{1}}} + {{\rm{\tau }}_{\rm{2}}} - {{\rm{\tau }}_{\rm{0}}}{\rm{)}} \nonumber \\ &&\quad - {\rm{\pi }}({{\rm{\nu }}_{\rm{1}}}) - {{\rm{\tau }}_2} - ({\rm{echo}}) \end{eqnarray*}

The 16–20 ns pump pulse at a microwave frequency *ν*_2_ was applied on the maximum of the nitroxide spectrum. The detection pulses were applied at a microwave frequency ν_1_ with an offset Δ*ν* = *ν*_2_ – *ν*_1_ = 55 MHz. Pulse sequences were generated by a Bruker arbitrary wave generator (AWG) using square pulses (14,15). The dipolar evolution time was chosen to be 2.5 μs. Longer evolution times (τ_2_) up to 4 μs did not affect the results of the experiments, but they have been tested to confirm the longest distance value within the different distance distributions. The separation time τ_1_ was set to 204 ns. A model-free analysis of DEER data was performed by with the Tikhonov regularization approach, using the L-curve as criterion for optimal parameter regularization. Primary experimental data were background-corrected by fitting a decay function *B*(*t*) for the intermolecular contribution, followed by normalization of the function. The model-free processing was performed with the program DeerAnalysis2018 (16–19).

### Protein expression and purification

MAX was subcloned into a Pet3d expression vector and transformed into *Escherichia coli* BL21 pLysS cells. Cells were grown at 37°C in M9 (for ^15^N labeling 1 g/l ^15^N ammonium chloride was added) and induced at an optical density corresponding to *A*(600 nm) = 0.5 with 0.5 mM IPTG prior to incubation at 30°C overnight. Cell pellets were homogenized in 20 mM PBS, 100 mM NaCl and 1 mM EDTA. For protein purification, fractional (NH_4_)_2_SO_4_ precipitation (50% and 80% saturation) was carried out and anion exchange chromatography was applied. The final total protein concentration was 0.4 mM.

Cysteine mutants and MTSL (*S*-(1-oxyl-2,2,5,5-tetramethyl-2,5-dihydro-1H-pyrrol-3-yl)methyl methanesulfonothioate) labeled proteins were produced according to methods published earlier (20,21). Excess spin label was removed by dialysis into the buffer used for the NMR experiments. The labeling efficiency was always >95% as determined via DTNB assays.

PRE referencing was achieved by reduction of the MTSL label through incubation for 1 h with tenfold excess of ascorbic acid at 35°C. For DEER EPR, samples were vitrified, i.e., flash frozen at their glass transition temperature by plunging the samples into liquid nitrogen after the addition of 15% glycerol to avoid crystallization. EBOX double stranded DNA oligos AAACACGTGAAA were purchased from Eurogentech. The EBOX DNA:MAX_2_ ratio was 1:1 in all experiments.

### Molecular dynamics simulations

To visualize the structural fluctuations of the NTD domain of the MAX_2_/DNA complex in solution, we performed molecular dynamics (MD) simulations using GROMACS 2018.3 (22). The crystal structure of a human MAX_2_/DNA complex (PDB ID: 1HLO) was used in our simulations to build our initial atomistic model. The complex was confined with 21750 water molecules in a dodecahedral box so that the edges of the box were always at least 1 nm away from the complex. The structure was electro-neutralized with Na^+^ ions, and Na^+^Cl^−^ ions were added to achieve a salt concentration of 25 mM as used in the experiments. NVT equilibration was performed at *P* = 1 bar and *T* = 310, 320 and 330 K in a constrained box for 80 ps, with a step of 2 fs using the Verlet cutoff scheme set to 1.2 nm. The modified Berendsen thermostat temperature scheme coupled the protein and non-protein thermostats. Subsequently, NPT equilibration was performed under similar conditions. Nosé-Hoover thermostat coupling was used, which allows wide fluctuations and produces more natural dynamics than the Berendsen coupling. AMBER03 and AMBER94 force fields were used for the protein and the DNA respectively (23). The MD simulations continued NPT equilibration under unconstrained conditions. The MD trajectories were sampled every 200 ps, for a total simulation time of 100 ns.

## RESULTS AND DISCUSSION

**Figure 1.**
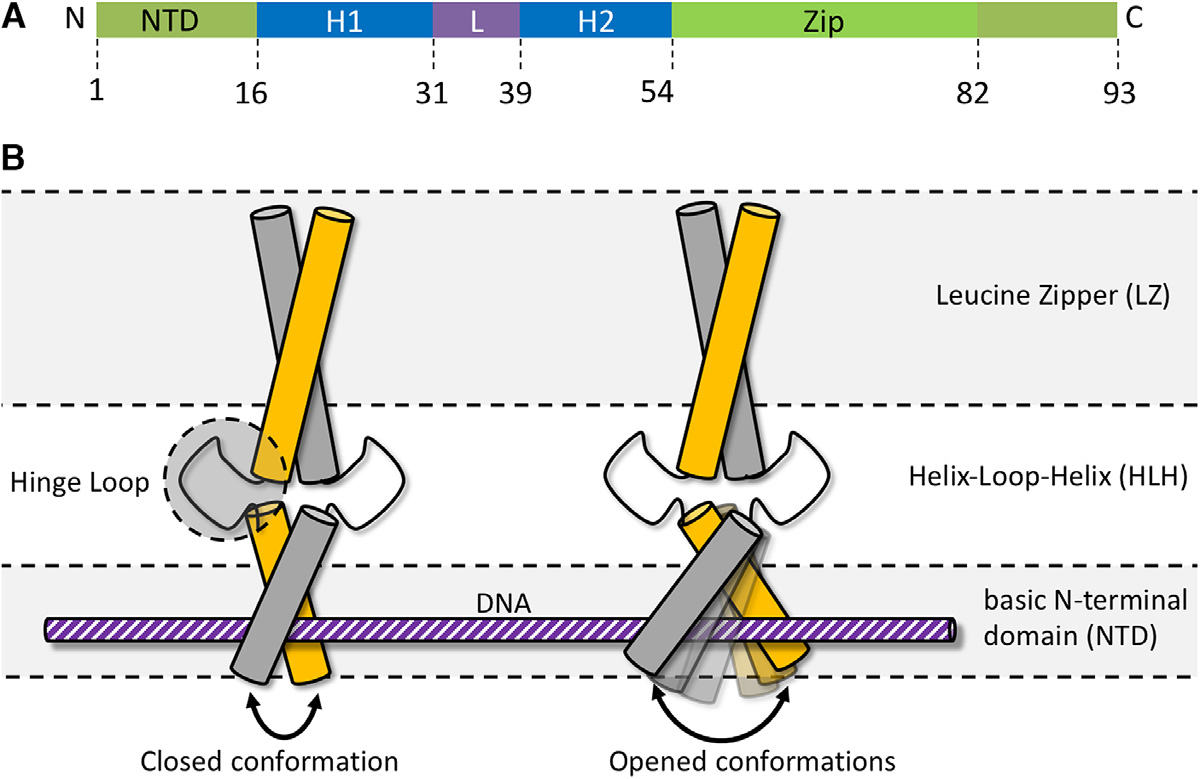
Schematic illustration of conformational fluctuations. (**A**) The MAX_2_ homodimer adopts a basic-helix-loop-helix-leucine zipper (b/HLH/LZ) conformation preceded by a basic N-terminal domain (NTD) that houses the DNA binding epitope (35,36). (**B**) Scheme of two limiting cases: compact, closed DNA-bound state of MAX_2_ (left) and open state (right) that features an increased distance between the two basic NTDs. The NTD samples a continuum of states with varying distances between the two subunits. The HLH motif serves as hinge between the LZ and basic domains. The different domains of MAX_2_ are indicated on the right.

Each subunit of the homodimer MAX_2_ can be dissected into three domains (Figure 1A): (i) a basic N-terminal domain (NTD) that houses the DNA binding epitope, (ii) a helix-loop-helix (HLH) motif that connects the NTD to (iii) a leucine zipper (LZ) that serves as anchor between the two subunits that form the coiled-coil homodimer. By undergoing a scissor-type motion, the size of the DNA binding cleft is modulated: the HLH domain acts as hinge between the rigid LZ and the NTD, which samples a continuum of states between an open and a closed conformation (see Figure 1B). We find that the opening of the binding cleft varies in width by ca. 2–5 nm. Such a distance corresponds well to fluctuations between two limiting cases: (i) an open structure loosely coordinating the EBOX DNA, and (ii) a compacted structure wrapped tightly around the ligand.

The first indications of such structural fluctuations were found *via* a paramagnetic relaxation enhancement (PRE) nuclear magnetic resonance (NMR) approach (24–26). These experiments revealed transiently formed contacts between the NTDs and the HLH segments of DNA-bound (holo)-MAX_2_. PRE NMR is a solution-state technique based on site-directed spin labeling (SDSL) (27–29), in which a paramagnetic side chain (spin label; SL) is attached to a selected residue of a protein. The SL increases relaxation rates for the NMR-active nuclear spins in its vicinity, resulting in reduced signal amplitudes for amino acids within a radius of ca. 2–3 nm around the SL. This NMR signal reduction can be quantified in a residue-resolved manner as the signal suppression ratio *V^i^* = *S*^i^_PRE_*/S*^i^_REF_ for the *i*th residue, where *S*^i^_PRE_ is the ^1^H–^15^N cross peak signal amplitude observed in the presence of the label and *S*^i^_REF_ is the corresponding amplitude in a reference spectrum obtained with a deactivated diamagnetic label. *V* follows a steep *r*^−6^ proportionality, where *r* is the distance between the SL and an observed amino acid, and depends also on the dynamics of the protein. A complete suppression of a signal (*V* = 0) always requires that *r* < 2.5 nm (see, e.g. the work by Wagner and co-workers for details (30)). Proximity measures are thus accessible between the labeling site and all other adjacent residues of a protein.

**Figure 2.**
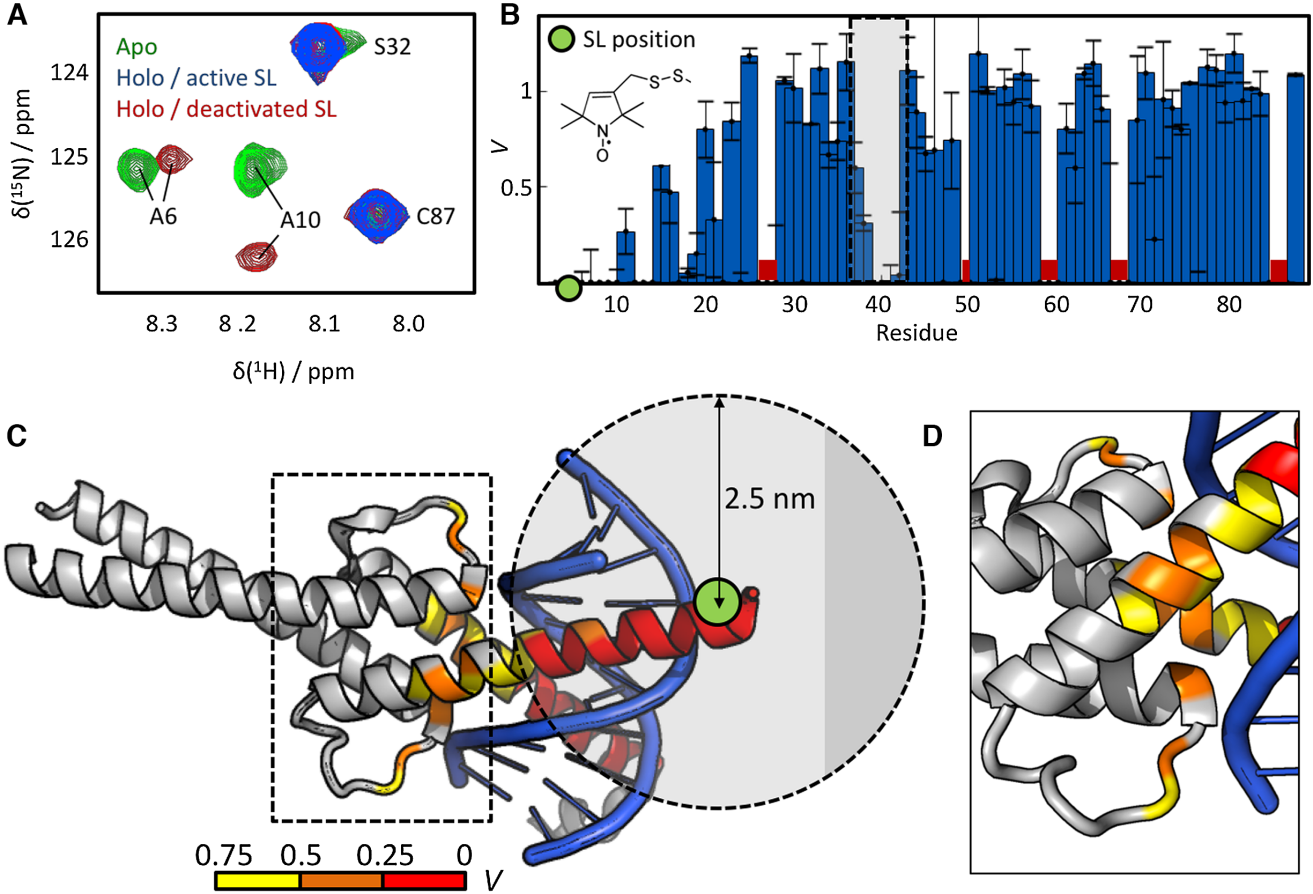
PRE NMR reveals conformational fluctuations of the MAX_2_/DNA complex. (**A**) Section of a ^1^H–^15^N HSQC of MAX_2_ in the DNA-free apo-state (green), the DNA-bound holo-state with an active SL attached to position R5C (blue) and in the holo-state with a deactivated, chemically reduced SL (red). No trace of residual apo-MAX_2_ is observed in the presence of DNA. Signals of residues located close to the labeling site are suppressed by the SL, while remote residues remain unaffected. (**B**) Residue dependence of the signal suppression ratio *V* between spectra with active and deactivated SL. Around the labeling site (green dot) signals are suppressed (*V* = 0 for residues 0–10) or reduced (*V* < 1 for residues 10–25). Signals between residues 38–42 (indicated by the gray shade) are likewise suppressed or reduced. The molecular structure of the MTSL label is indicated. Red bars indicate residues that were excluded from the analysis due to weak signals or overlap. (**C**) The intensity ratios *V* are mapped onto the crystal structure of MAX_2_/DNA (PDB entry 1HLO; for a DNA-free NMR-derived solution structure see PDB entry 1R05). The color code is indicated at the bottom. Residues 38–42, located in the HLH motif (indicated by the dashed box), are >2.5 nm distant from the labeling site (green dot). Contrary to the experimental observation, no strong signal reductions would therefore be anticipated for these residues based on the crystal structure analysis. (**D**) Zoom on the dashed box in (C). The reduced intensity ratio *V* in the HLH region indicates conformational fluctuations that lead to reduced distances between the SL and the HLH motif.

To explore the dynamics of the DNA binding epitope of MAX_2_ we produced an R5C mutant of MAX_2_ and selectively introduced a SL (the nitroxide MTSL; see the Supplementary Material) at position 5, i.e. within the NTD, of each subunit. This resulted in a doubly spin-labeled MAX_2_ denoted henceforth as SL-R5C-MAX_2_ (cf. Figure 1A). The effect of the SL was then studied by ^1^H–^15^N correlation NMR (see Figure 2a and Figure S1 of the Supplementary Material for the full spectrum). Two conclusions followed from the spectra: (i) The spectra of the DNA-free (apo; green) and DNA-bound (holo; red) states are clearly distinct, which, considering the slow exchange between the apo- and holo-forms (31), indicates that no free MAX_2_ was present during our experiments. This is in accordance with the low DNA-dissociation constant of *K*_D_ ≈ 10^−18^ M^2^ (31), which similarly points towards very high DNA affinity. (ii) Introduction of the SL leads to a disappearance of NMR signals for residues close to the labeling site. Figure 2B visualizes the residue-dependence of the signal suppression ratio *V^i^* for the entire protein. Both MAX_2_-subunits are identical, hence, each residue index corresponds to two equivalent amino acids. Proximate to the labeling sites, signals are clearly either entirely suppressed (*V* = 0 for residues 0–10) or reduced (*V* < 1 for residues 10–25).

Additionally, signals in the HLH domain, between positions 38 and 42 (indicated by the grey shade) are also affected. Notably, the signal of residue 41 is even reduced to naught indicating that holo-MAX_2_ samples conformations with distances *r* < 2.5 nm between this residue in the HLH domain and residue R5C in the NTD. This finding is unexpected, as a previous crystal structure analysis (2) of MAX_2_ bound to DNA suggested a distance of > 2.8 nm between the labeling site and position 41, hence, in contrast to the PRE NMR result. (The crystal structure of the DNA complex is henceforth denoted as MAX_2_/DNA to distinguish it from holo-MAX_2_ as used in our experiments.) Figure 2C and D map the observed suppression ratios *V* onto the MAX_2_/DNA crystal structure and indicate the position of the labeling site. The HLH domain is seen to lie outside the distance window of 2.5 nm around the SL, in which signals would be completely suppressed.

Hence, the NMR results can only be rationalized by considering structural fluctuations of holo-MAX_2_ in solution that lead to transient deviation from the structure it adopts in a crystal.

To further understand these structural fluctuations, we employed DEER (double electron-electron resonance, also known as pulsed electron double resonance; PELDOR) EPR (electron paramagnetic resonance) spectroscopy distance measurements (14). In DEER experiments one flash freezes, i.e., vitrifies a protein solution at its glass transition temperature (see the Supplementary Material for details) and measures the distribution *P*(*r*) of distances *r* between two simultaneously attached SLs (32). Through this, the presence of several co-existing conformations of a protein can be determined on length scales of ca. 1.5 < *r* < 10 nm, thereby revealing structural fluctuations within this range. The distance distribution *P*(*r*) represents the dynamics of the system under investigation, as it reflects a snapshot of the conformational ensemble at the time of sample vitrification. This approach revealed that the NTD of holo-MAX_2_ samples a continuum of conformations ranging from very compact, with the two NTDs separated by only 2 nm and wrapped tightly around the target DNA, to expanded, with distant NTDs separated by more than 4 nm (cf. Figure 1B).

**Figure 3.**
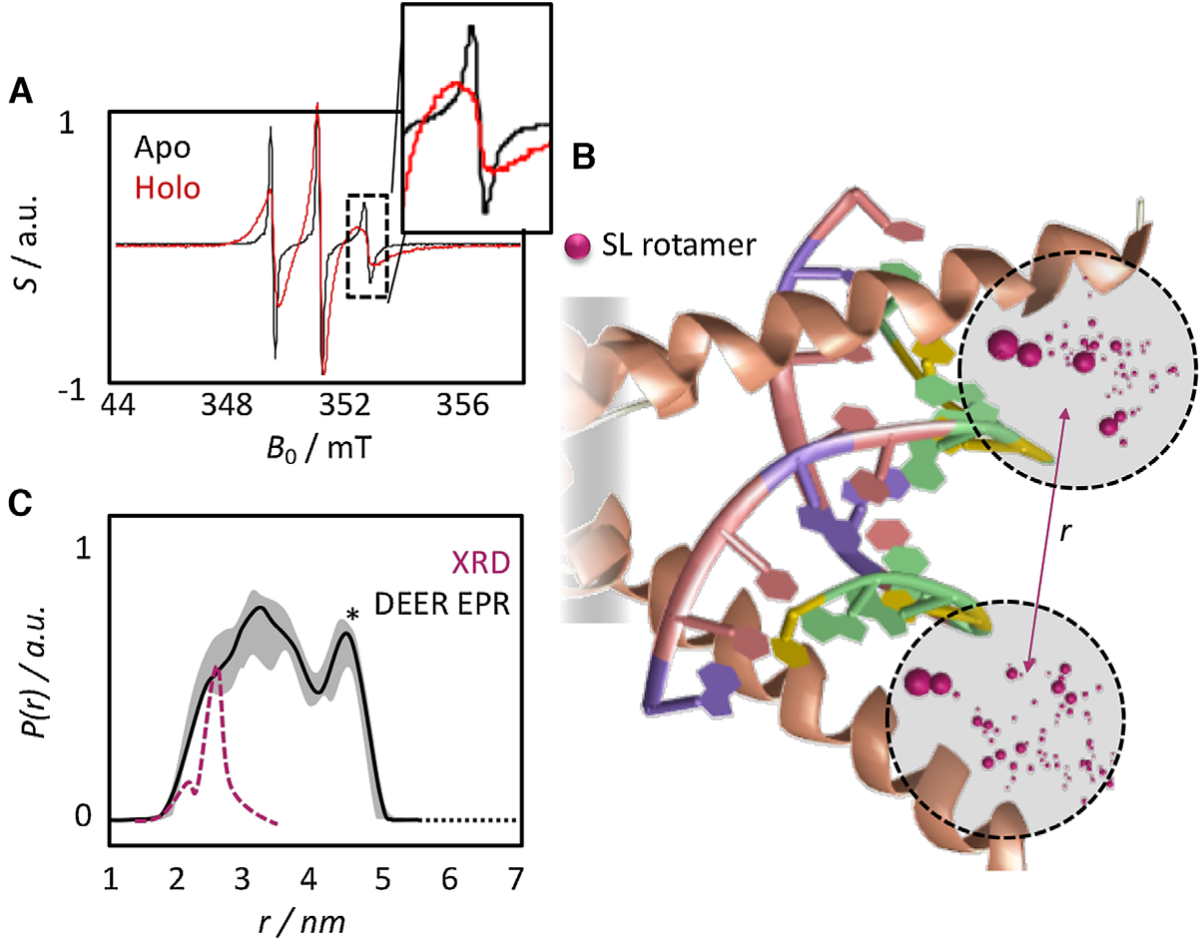
EPR reveals a conformational space with closed and opened DNA binding epitopes. (**A**) CW EPR of DNA-free apo-state (black; narrow signals), the DNA-bound holo-state (red; broad signals) of the SL-R5C mutant. Apo-MAX_2_ leads to sharp signals, which cannot can be observed in the presence of DNA. The insert shows a zoom on the high-field transition, where the difference between holo- and apo-state is most pronounced. (**B**) Graphical representation of the rotamer distribution of the two SL in the DNA-bound R5C mutant based on the crystal structure of MAX_2_/DNA (PDB code: 1HLO). Purple spheres indicate the position of the unpaired electron in the MTSL labels predicted by the MMM software (see main text). Relative sizes correspond to relative populations of the respective rotamers. (**C**) Experimental (black) and MMM-predicted (purple) distance distribution *P*(*r*) between the two spin labels in SL-R5C MAX_2_. The crystal structure (XRD) analysis yields a sharp distribution centered ∼2.5 nm. The experiment in vitrified solution yields a broad distribution indicating a continuum of co-existing conformations with distances between the two labeling sites varying between 2 and 5 nm. This indicates that the NTD samples states between two limiting cases, an open and a closed conformation around the bound DNA strand. The error is indicated as grey shade. The peak marked with the asterisk might be subject to uncertainties (see the supplementary material).

In room-temperature continuous-wave (CW) EPR experiments, DNA-free MAX_2_ gives rise to very sharp, narrow signals. This was clearly not observed prior to vitrification in the presence of DNA (Figure 3A) indicating the absence of any significant amounts of DNA-free (apo) SL-R5C-MAX_2_ in our samples.

We then measured the distribution *P*(*r*) between the two labels of the DNA-bound SL-R5C-MAX_2_ mutant. Figure 3B localizes possible SL positions expected according to the crystal structure of MAX_2_/DNA and Figure 3C displays the experimentally obtained distance distribution *P*(*r*). A continuum of distances *r* between the two labelling sites was observed spanning ca. 2–5 nm. Clearly, to give rise to the distribution seen in Figure 3C, the structural ensemble of holo-MAX_2_ in solution must include various conformations with varying distances between the two SLs, i.e., between the two NTDs, which are trapped upon vitrification (see Supplementary Figures S2 and S3 for the raw data and details on the data treatment).

To interpret this finding and compare it to existing structural models, we predicted a distance distribution *P*(*r*) based on the crystal structure of MAX_2_/DNA. We employed the MMM software (24), which predicts a set of rotamers for the two SLs and estimates the SL–SL distance for each rotamer pair. (For details see the work by Jeschke *et al.* (33).) The purple spheres in Figure 3B visualize the computed rotamer distributions for the two MTSL labels in SL-R5C-MAX_2_.

The predicted distribution obtained by this procedure is superimposed as a purple dashed line over the experimental result in Figure 3C. The prediction displays a narrow *P*(*r*) centered ∼2.5 nm, which coincides with the shortest distances that were experimentally determined. In other words, the predicted *P*(*r*) represents the compressed conformation that MAX_2_/DNA adopts in the crystal, in which the DNA binding epitope is wrapped tightly around the EBOX motif. In stark contrast, the DEER experiment unambiguously shows that substantial conformational plasticity is a dynamic solution-state feature of the NTD of MAX_2_ even in the holo-form. The conformational space ranges from the compacted NTD found in the crystal to an enlarged state with an almost doubled spatial extension of the DNA binding epitope beyond 4 nm. This represents remarkable new information about such an important transcription factor, and clearly provides an important complement to crystal structure analyses of TFs.

Note that distance distributions for SLs in the HLH (SL attached to position G35C) and LZ (SL attached to position R55C, cf. Figure 1) domains are not significantly affected by binding to EBOX DNA. (See the Supplementary Material. Figures S4–S7 contain the raw and processed DEER data for the apo- and holo-states of the HLH and LZ domains.) The interaction with the EBOX DNA is therefore found to only influence the structural dynamics of the NTD. Additionally, a rotamer analysis of SLs at positions G35C and R55C showed that the solution conformations of the HLH and LZ domains are well-described by the crystal structure of MAX_2_/DNA (see Supplementary Figures S5 and S7).

**Figure 4.**
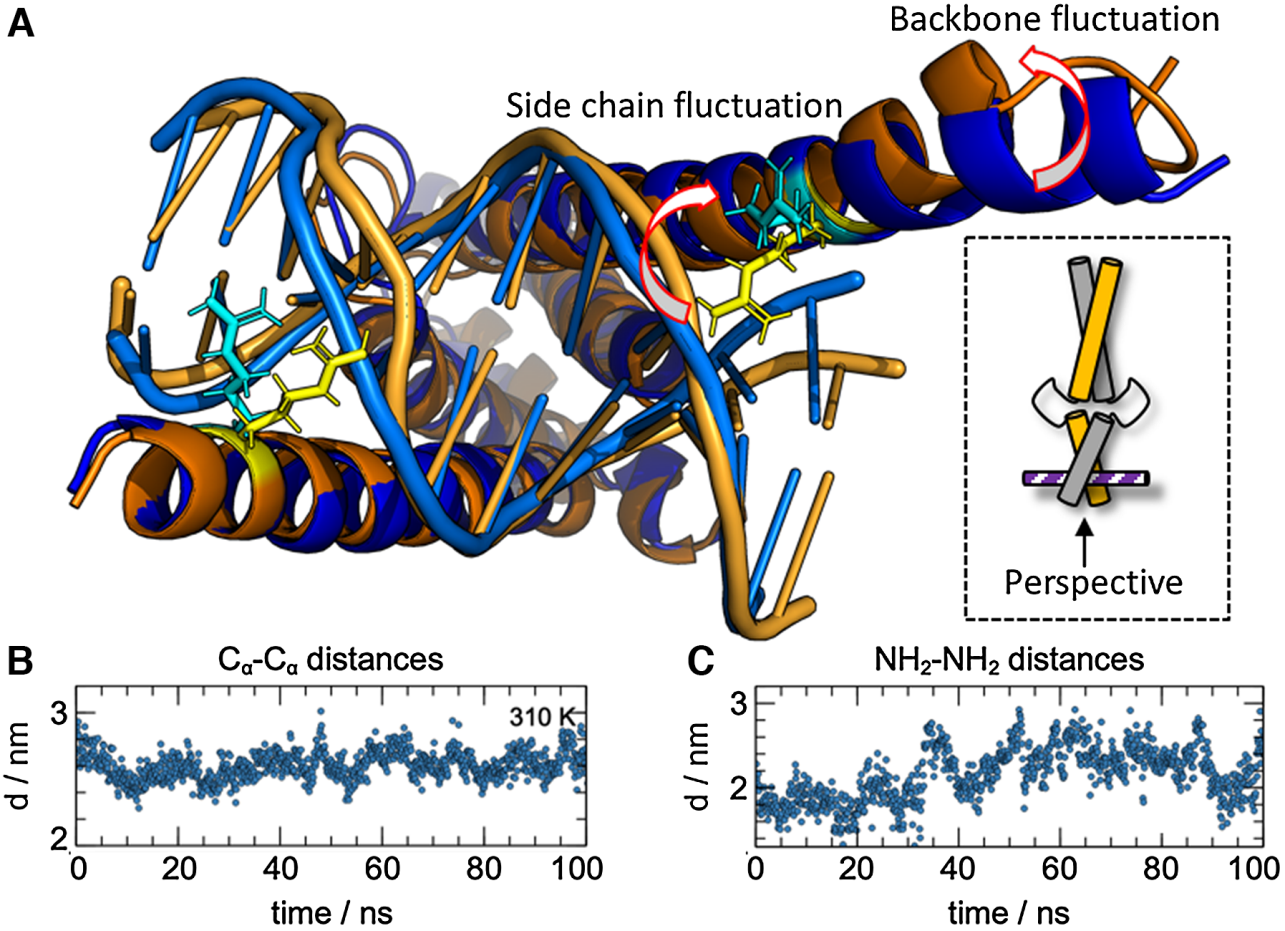
MD simulations visualize opened and closed conformations of the NTD of holo-MAX_2_. (**A**) Representative structures for opened (orange) and closed (blue) conformations sampled in our MD simulations. The arrows indicate how the side chains of residues R5 wrap around the DNA in the closed conformation, but release it in the open conformation, and how the helical backbone loosens from the bound DNA ligand. (**B**) C_α_–C_α_ distances, representative of the separation between the backbones of the two helices, for residues R5 (the MTSL labeling sites) during the MD run for each time frame in a 100 ns simulation at 310 K. (**C**) NH_2_–NH_2_ distance, representative for the separation between side chains, for residues R5 (the MTSL labeling sites) during the MD run for each time frame in a 100 ns simulation at 310 K.

Finally, to visualize and further confirm the structural fluctuations of the NTD, we performed molecular dynamics (MD) simulations. (For details, see the Materials and Methods section and the Supplementary Material.) The simulations corroborate the experimentally observed conformational sampling between closed and opened conformations. Several MD trajectories at 310, 320 and 330 K displayed significant structural fluctuations within the NTD of holo-MAX_2_ as it continuously expands and closes around the bound EBOX DNA double strand. The HLH motif acts as hinge between the LZ and NTD domains, while the LZ forms a rigid anchor between the two MAX monomer units. Figure 4A visualizes the different conformations sampled in our simulations and emphasizes the opening (orange) and closing (blue) of the NTD. The simulations further indicated in addition to the fluctuations of the two helices that form the backbone of the NTD, an unfolding of the side chains of residue R5 away from the DNA ligand also correlated with the sampling of the opened MAX_2_ conformation. The side chains wrap around the DNA ligand in the compacted form but release their grip in the widened conformation.

The simulated kinetics of the conformational switching between opened and closed form are visualized in Figure 4B and C, which show the variations of C_α_–C_α_ (backbone) and NH_2_–NH_2_ (side chain) distances between residues R5 (i.e. the labeling site in the PRE and DEER experiments) for a period of 100 ns at a temperature of 310 K. (For other temperatures, see the Supplementary Material Figures S8 and S9.) In the time traces, longer distances (*d*(C_α_–C_α_) ≈ 2.7 nm, *d*(NH_2_–NH_2_) ≈ 3 nm) correspond to the sampling of opened conformations, while shorter distances (*d*(C_α_–C_α_) ≈ 2 nm, *d*(NH_2_–NH_2_) ≈ 1.5 nm) correspond to sampling of closed conformations. Thus, the DNA binding mode is dynamically tuned by conformational switching on a nanosecond timescale, as MAX_2_ exchanges between a loosely bound, possibly energetically excited state and a tightly coordinated ground state.

Note that the MD simulations might fail to sample conformational exchange processes on longer (>100 ns) timescales due to their limited duration. Nevertheless, an analysis of rotamer distributions in the simulated structures confirmed that the combination of the opened and closed conformations can account for the experimental DEER data (see the Supplementary Material Figure S10).

## CONCLUSION

MAX_2_ is a model system for many b/HLH/LZ-based TFs. Our results show that this important molecule displays significant conformational plasticity within its DNA-binding epitope even in the holo-state. This is particularly interesting considering the recent descriptions of facilitated dissociation and facilitated diffusion:

Facilitated dissociation requires the sampling of energetically excited states in the DNA-bound form that reduce the binding energy that needs to be overcome for dissociation of the TF and the DNA—a prerequisite for molecular recognition processes like the MYC:MAX heterodimerization remote from the DNA strand. The open conformations sampled by the DNA-binding epitope of MAX_2_ may feature the required lower binding energy as a result of loosened contacts between ligand and host.

The opened conformations of MAX_2_, might foster facilitated diffusion along the DNA. The transiently reduced binding energy would enhance MAX_2_’s activity despite its very low *K*_D_ of ca. 10^−18^ M^2^, by enabling accelerated translation along DNA strands. Such an open conformation would correspond to a loosely bound ‘search state’ of the TF that rapidly samples the DNA, while the closed conformation would represent a ‘recognition state’ that specifically identifies the cognate DNA motif (7,31,34).

## DATA AVAILABILITY

All data are available in the Zenodo online repository under DOI 10.5281/zenodo.2628267.

## Supplementary Material

gkz291_Supplemental_File
