# Supplementary material for: Conformational tuning of a DNA-bound transcription factor
Source: Nucleic Acids Res. 2019 Apr 25;47(10):5429–35. doi: 10.1093/nar/gkz291 (PMC6547406; doi:10.1093/nar/gkz291)
Supplement: gkz291_Supplemental_File [file gkz291_supplemental_file.pdf]

Supplementary Data

Supplementary NMR Data

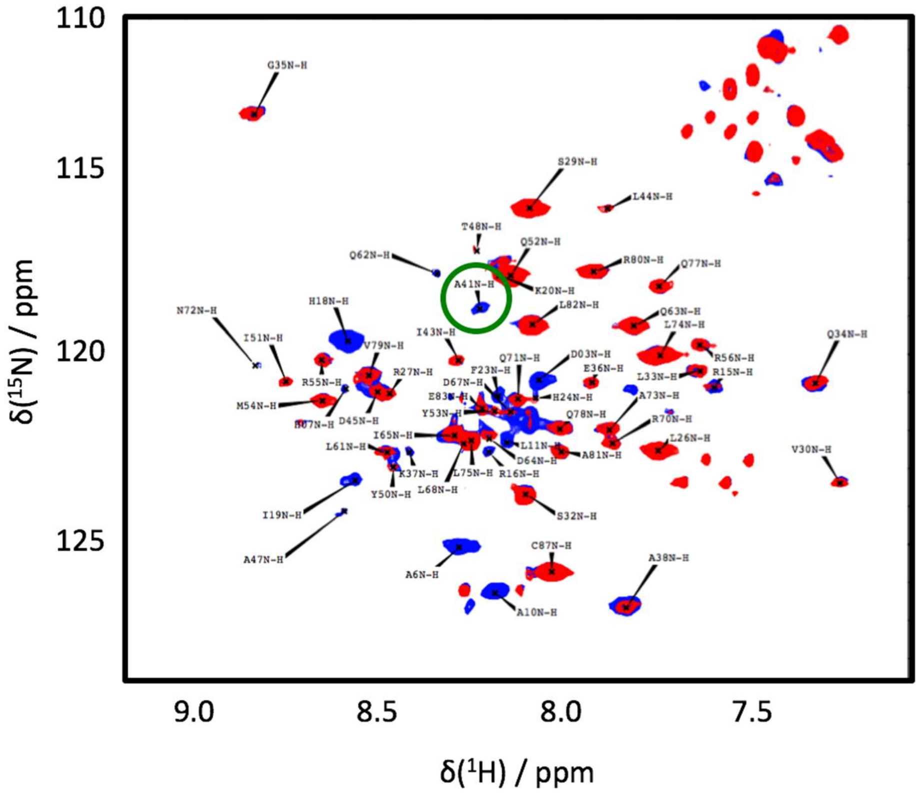

Figure S1. ^{1}H-^{15}N TROSY of holo SL-R5C-MAX$_2$ (red), and holo SL-R5C-MAX$_2$ with a reduced SL (blue), at a magnetic field of 18.8 T and a temperature of 293 K. The amino acid assignments are indicated. Residue A41, which is located in the HLH domain, is highlighted by the green circle. The signal for this residue is entirely suppressed in the presence of the SL indicating contacts between residue R5C and A41.

Supplementary DEER EPR data

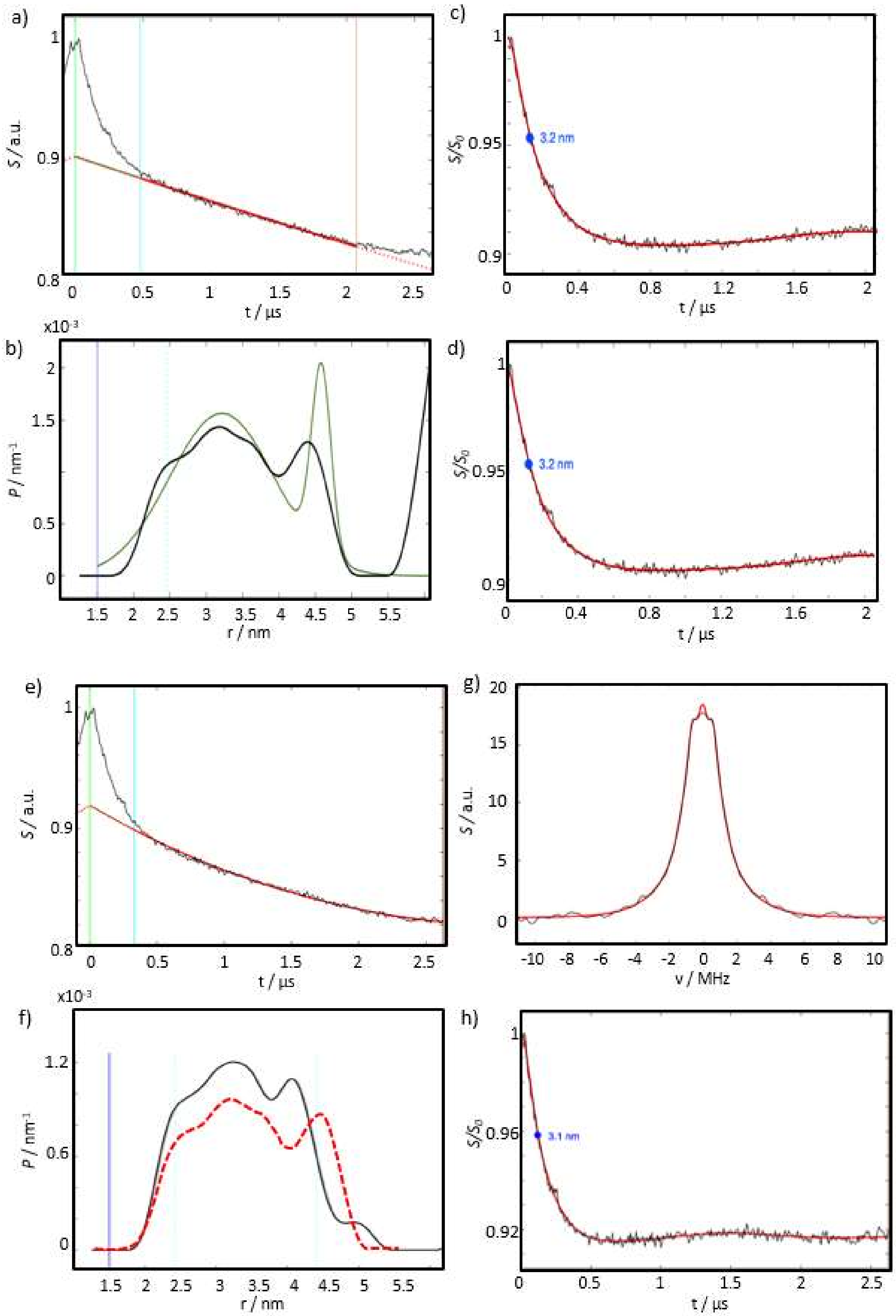

Figure S2. a) Raw DEER data for holo-MAX₂. The red line indicates the fitted background function. The echo's zero time is indicated by the green line. b) Distance distributions obtained from the data in panel a) by a Tikhonov

regularization (black) and a model fit with 2 Gaussian distributions (green) of the DEER time domain trace of holo SL-R5C-MAX2. All distances present in the Tikhonov approach also appear in the model fit supporting the reliability of the obtained distances. c) Fit by Tikhonov regularization (red) and background corrected time trace, i.e., form factor (black). d) Fit by 2 Gaussians (red) and background corrected time domain trace (black). e) Same as in a), but the background was fitted to a second-order polynomial. f) Distance distribution obtained from Thikhonov regularization of the data in e) (black, 2nd order polynomoial background model) superposed with the distance distribution from panel b) (red dashed line, homgeneous background model). Only the maximum at 4 nm, 4.5 nm, respectively, is affected by changing the background model demonstrating the robustness of the obtained distance distribution for distances below 4 nm. g) Dipolar spectrum (black) and fit (red) after correction of the data with the 2nd order polynomoial background model. h) Corresponding form factor (black) and fit (red) after correction of the data with the 2nd order polynomoial background model.

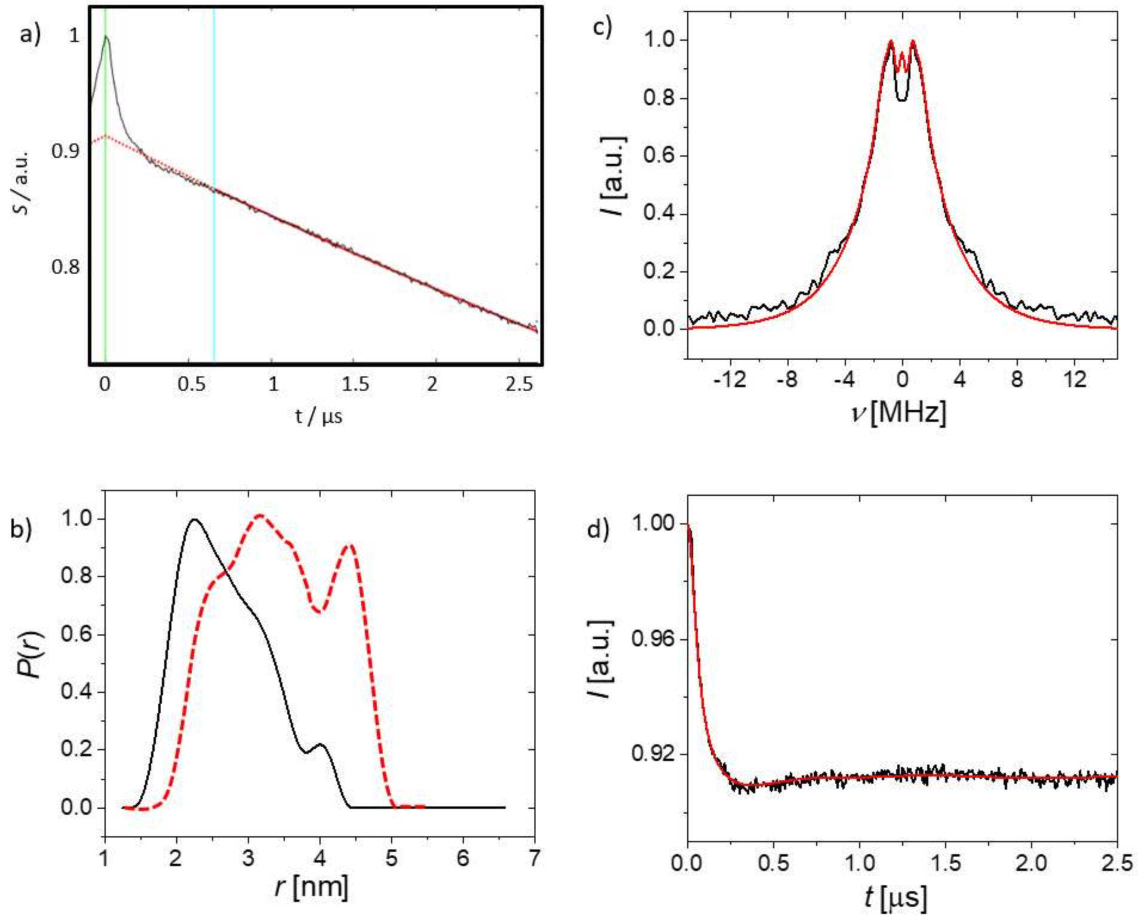

Figure S3. a) Experimental DEER time trace (black) and homogeneous background fit (red) for apo-SL-R5C-MAX2. b) Distance distribution *P(r)* for SL-R5C-MAX2 in the apo- (black) obtained from the data in panel (a) by the Thikonov regularization approach, superimposed on the distribution found in the holo-state (dashed red line, same as in Figure S1f. c) Dipolar spectrum for SL-R5C-MAX2 in the apo-state (black) and corresponding fit (red). d) Form factor after background correction (black) and corresponding fit leading to the distribution in (b) (red).

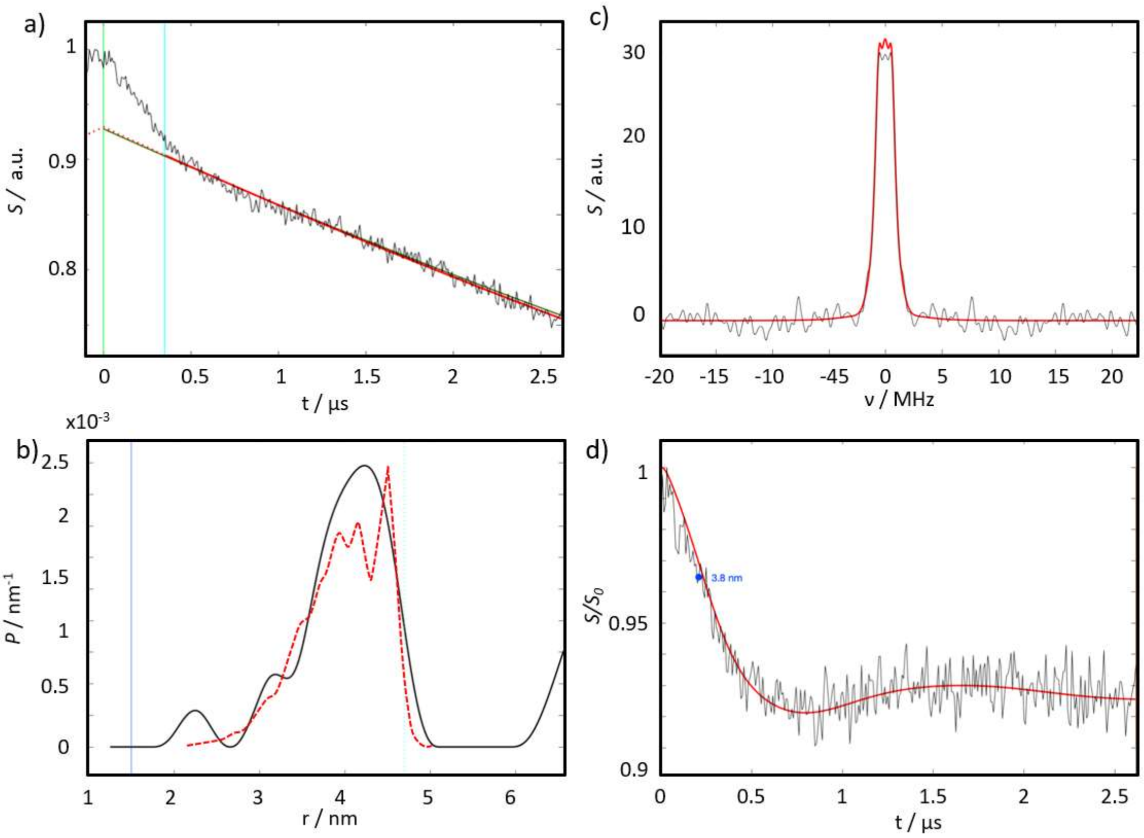

Figure S4. a) DEER signal of SL-G35C-MAX$_2$ labeled at position 35 located in the HLH domain in the holo-state. The red line indicates the fitted homogeneous background function. The green line indicates the center of the echo. b) Corresponding distance distribution *P(r)* (black). The red dashed line shows an MMM prediction from a rotamer analysis of the crystal structure (PDB entry 1HLO). c) Dipolar spectrum (black) and fit (red) leading to the distance distribution in (b). d) Form factor after background correction (black) and fit (red) leading to the distance distribution in (b).

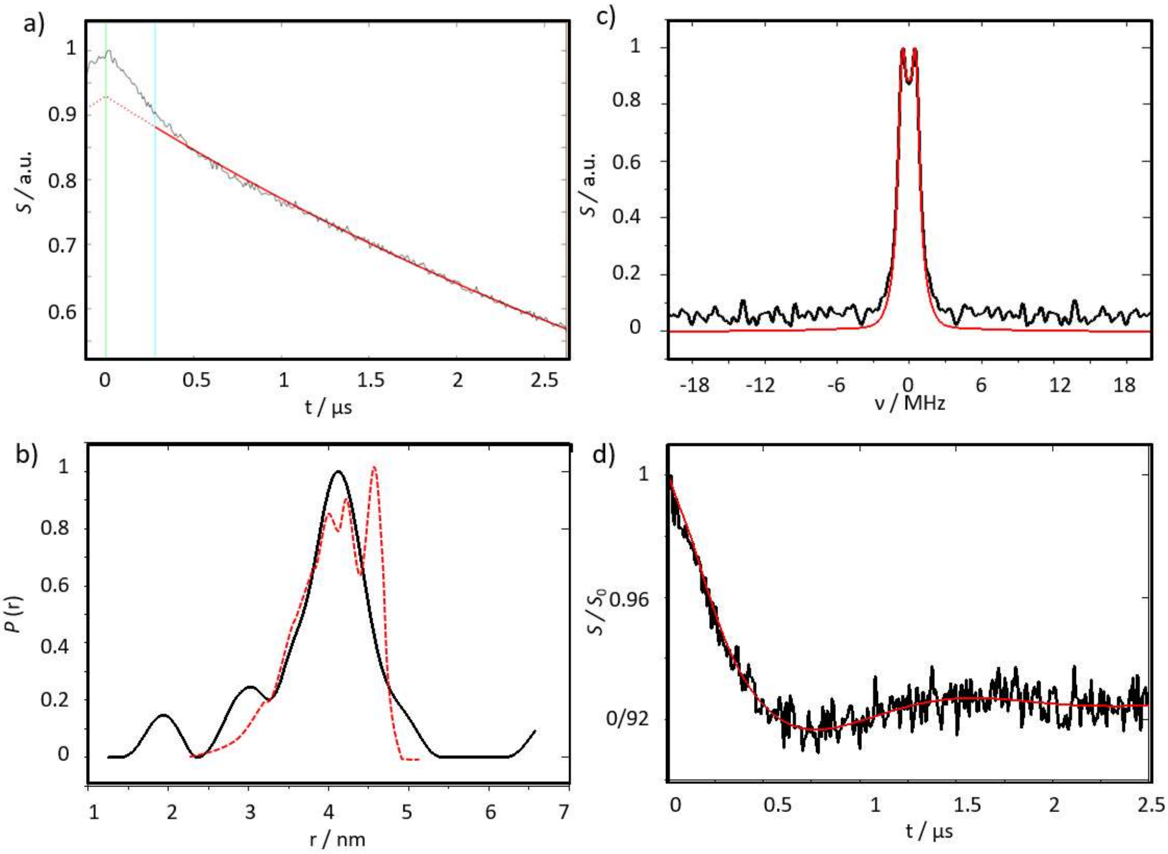

Figure S5. a) DEER signal of SL-G35C-MAX_2 labeled at position 35 located in the HLH domain in the apo-state. The red line indicates the fitted homogeneous background function. The green line indicates the center of the echo. b) Corresponding distance distribution *P(r)* (black). The red dashed line shows an MMM prediction from a rotamer analysis of an NMR solution structure (PBD entry: 1R05). c) Dipolar spectrum (black) and fit (red) leading to the distance distribution in b). d) Form factor after background correction (black) and fit (red) leading to the distance distribution in b).

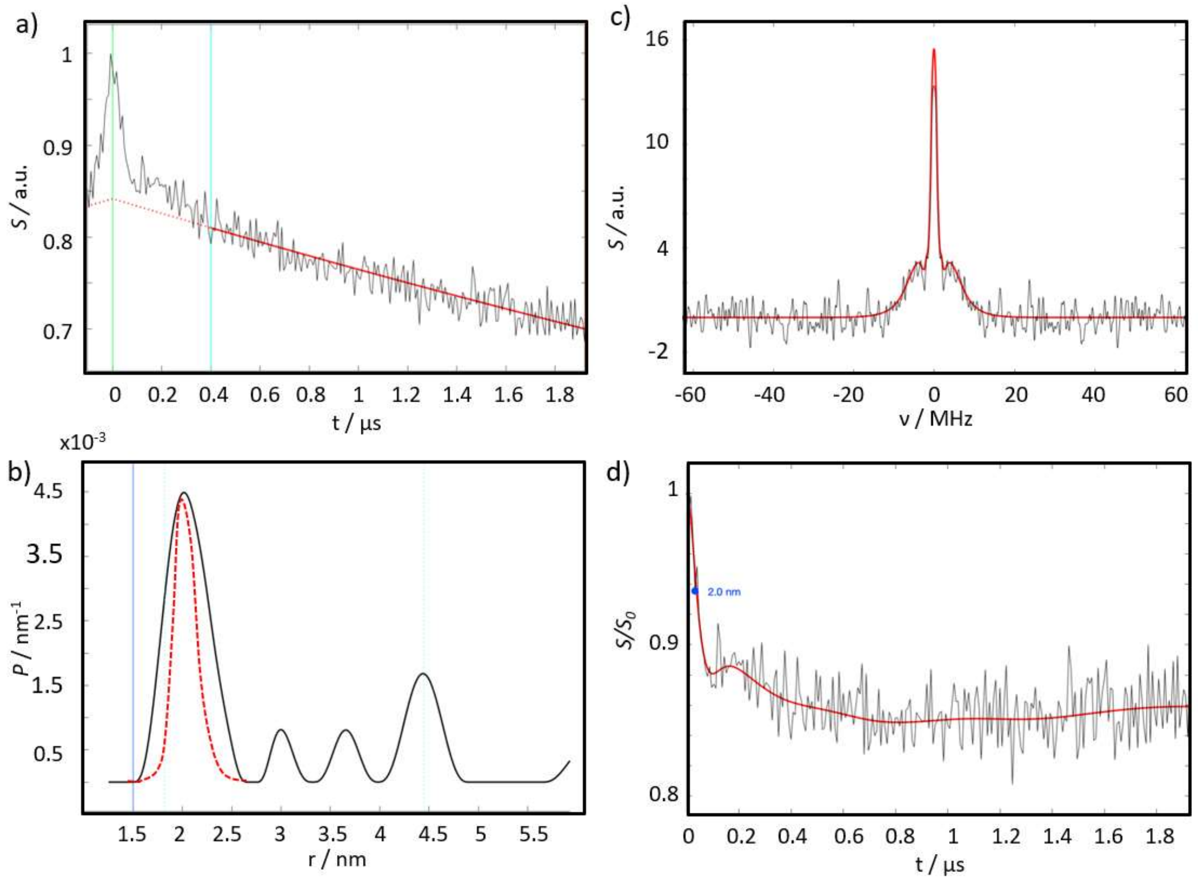

Figure S6. a) DEER signal of SL-R55C-MAX$_2$ labeled at position 55 located in the LZ domain in the holo-state. The red line indicates the fitted homogeneous background function. The green line indicates the center of the echo. b) Corresponding distance distribution *P(r)* (black). The red dashed line shows an MMM prediction from a rotamer analysis of the crystal structure (PDB code 1HLO). c) Dipolar spectrum (black) and fit (red) leading to the distance distribution in (b). d) Form factor after background correction (black) and fit (red) leading to the distance distribution in (b).

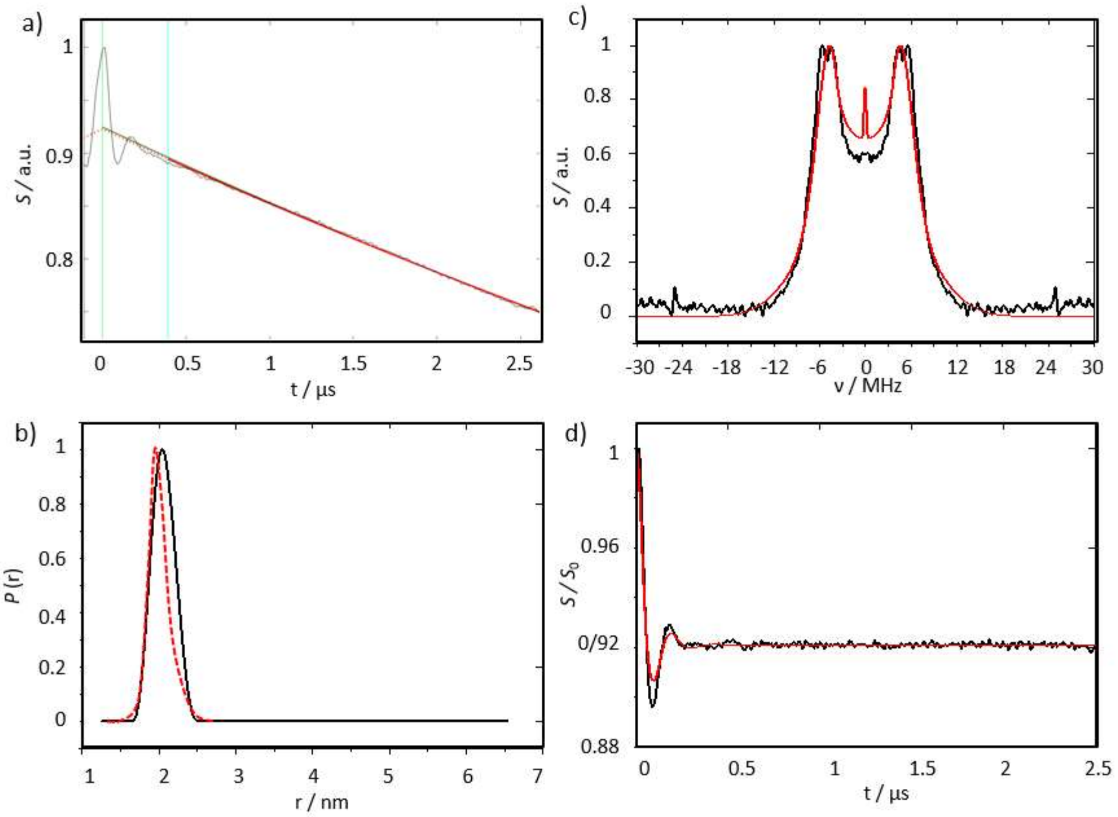

Figure S7. a) DEER signal of SL-R55C-MAX$_2$ labeled at position 55 located in the LZ domain in the apo-state. The red line indicates the homogeneousfitted background function. The green line indicates the center of the echo. b) Corresponding distance distribution *P(r)* (black). The red dashed line shows an MMM prediction from a rotamer analysis of the NMR solution structure (PDB code: 1R05). c) Dipolar spectrum (black) and fit (red) leading to the distance distribution in (b). d) Form factor after background correction (black) and fit (red) leading to the distance distribution in (b).

Comparison of the MD simulations with experimental data

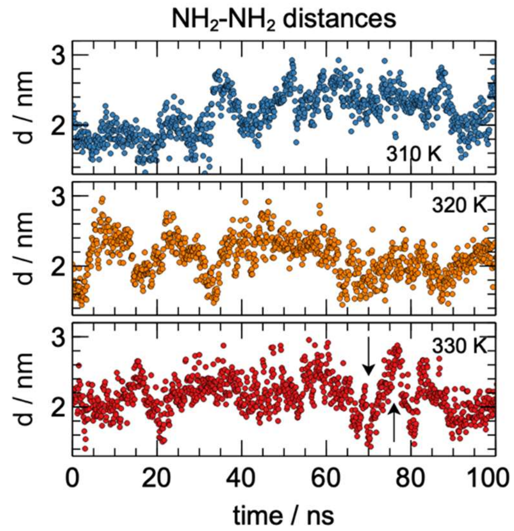

Figure S8. NH_2-NH_2 distances, representative of the separation between the backbones of the two helices, for residue R5 (the MTSL labelling site) during the MD run for each time frame in a 100 ns simulation at 310, 320 and 330 K. At all three temperatures, backbone distances within the NTD fluctuate between ca. 1.5 and 3 nm.

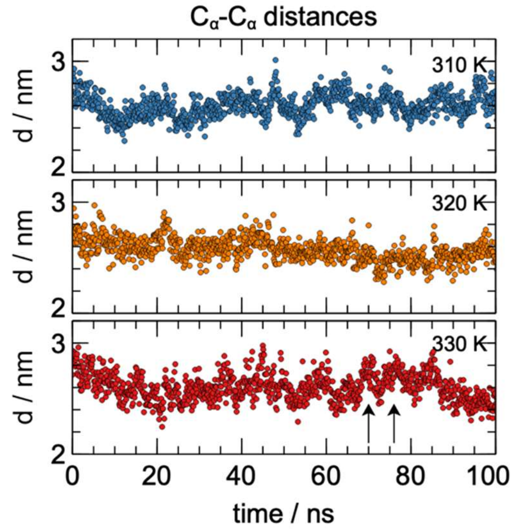

Figure S9. C_α-C_α distances, representative of the separation between the backbones of the two helices, for residue R5 (the MTSL labelling site) during the MD run for each time frame in a 100 ns simulation at 310, 320 and 330 K. Here, it is seen that at all three temperatures, backbone distances within the NTD fluctuate between ca. 2 and 3 nm.

Note that the simulations sample only NH_2-NH_2 distances between 1.5 and 3 nm, while the experimentally determined distance distributions spans 2-5 nm. The rotamer analysis (Fig. S10) of the MD data, shows that the SL samples a wider space (with distances between 2 and 4.5 nm) than the side chain of an unmutated residue R5.

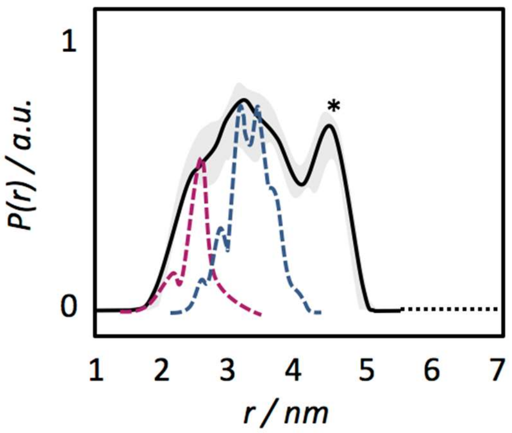

Figure S10. Experimental DEER R5-R5 distance distribution (back) superimposed on distance distributions calculated by a rotamer analysis of a closed MAX_2 conformation (purple; see main text) and an open MAX_2 conformation (blue) as obtained by the MD simulations of the MAX_2-EBOX DNA complex.

Evidently from Figure S10, the combination of the closed and opened conformations can account for the experimental distance distribution on a range between 2 and 4.5 nm. The distance peak indicated by the asterisk is not reproduced by the rotamer analysis of the open conformation. However, this is not surprising, as for longer distances (> 4 nm) DEER-derived distance distributions are prone to significant errors. Indeed, Fig. S2f illustrates how the position of the peak marked with the asterisk depends on the type of background correction used.
